# Organochlorine Compounds and Ultrasound Measurements of Fetal Growth in the INMA Cohort (Spain)

**DOI:** 10.1289/ehp.1408907

**Published:** 2015-06-09

**Authors:** Maria-Jose Lopez-Espinosa, Mario Murcia, Carmen Iñiguez, Esther Vizcaino, Olga Costa, Ana Fernández-Somoano, Mikel Basterrechea, Aitana Lertxundi, Mònica Guxens, Mireia Gascon, Fernando Goñi-Irigoyen, Joan O. Grimalt, Adonina Tardón, Ferran Ballester

**Affiliations:** 1 Foundation for the Promotion of Health and Biomedical Research in the Valencian Region, FISABIO-Public Health, Valencia, Spain; 2 Spanish Consortium for Research on Epidemiology and Public Health (CIBERESP), Spain; 3 Nursing Department, School of Nursing, University of Valencia, Valencia, Spain; 4 Department of Preventive Medicine and Public Health, University of Oviedo, Spain; 5 Department of Environmental Chemistry, Institute of Environmental Assessment and Water Research (IDÆA-CSIC), Barcelona, Spain; 6 Department of Surgery and Cancer, Imperial College London (UK), London, United Kingdom; 7 Department of Epidemiology, Public Health Division of Gipuzkoa, Basque Government, Gipuzkoa, Spain; 8 Health Research Institute, Biodonostia, San Sebastian, Spain; 9 Preventive Medicine and Public Health Department, University of the Basque Country, Bilbao, Spain; 10 Center for Research in Environmental Epidemiology (CREAL), Barcelona, Catalonia, Spain; 11 Pompeu Fabra University, Barcelona, Catalonia, Spain; 12 Department of Child and Adolescent Psychiatry/Psychology, Erasmus University Medical Centre–Sophia Children’s Hospital, Rotterdam, the Netherlands; 13 Public Health Laboratory, Basque Government, San Sebastian, Spain

## Abstract

**Background:**

Several studies have reported decreases in birth size associated with exposure to organochlorine compounds (OCs), but uncertainties remain regarding the critical windows of prenatal exposure and the effects on fetal body segments.

**Objective:**

We examined the relationship between prenatal OC concentrations and fetal anthropometry.

**Methods:**

We measured 4,4´-dichlorodiphenyldichloroethylene (4,4´-DDE), hexachlorobenzene (HCB), and polychlorinated biphenyl (PCB) congeners (138, 153, and 180) in 2,369 maternal and 1,140 cord serum samples in four Spanish cohorts (2003–2008). We used linear mixed models to obtain longitudinal growth curves for estimated fetal weight (EFW), abdominal circumference (AC), biparietal diameter (BPD), and femur length (FL) adjusted by parental and fetal characteristics. We calculated standard deviation (SD) scores of growth at 0–12, 12–20, and 20–34 weeks of gestation as well as size at gestational week 34 for the four parameters. We studied the association between OCs and the fetal outcomes by cohort-specific linear models and subsequent meta-analyses.

**Results:**

PCBs were associated with a reduction in AC up to mid-pregnancy, and BPD and FL from gestational week 20 onward. An inverse association was also found between HCB and AC growth in early pregnancy. The reduction of these parameters ranged from –4% to –2% for a doubling in the OC concentrations. No association between 4,4´-DDE and fetal growth was observed.

**Conclusions:**

To our knowledge, this is the first study to report an association between prenatal exposure to some PCBs and HCB and fetal growth: AC during the first two trimesters of pregnancy, and BPD and FL later in pregnancy.

**Citation:**

Lopez-Espinosa MJ, Murcia M, Iñiguez C, Vizcaino E, Costa O, Fernández-Somoano A, Basterrechea M, Lertxundi A, Guxens M, Gascon M, Goñi-Irigoyen F, Grimalt JO, Tardón A, Ballester F. 2016. Organochlorine compounds and ultrasound measurements of fetal growth in the INMA cohort (Spain). Environ Health Perspect 124:157–163; http://dx.doi.org/10.1289/ehp.1408907

## Introduction

Fetal growth is an important indicator of child health because its impairment may be associated with poor neurodevelopment (Richards et al. 2002) and with chronic diseases in adulthood (Barker 2007). It has been hypothesized that some organochlorine compounds (OCs) can cross the placenta (Vizcaino et al. 2014a) and may interfere with fetal development and growth (Windham and Fenster 2008).

To date, most studies on prenatal exposure to OCs and fetal growth have used anthropometric measures at birth (El Majidi et al. 2012; Govarts et al. 2012) and, to a lesser extent, outcomes such as being small for gestational age (Ribas-Fitó et al. 2002) as proxy measures of *in utero* growth. A limitation with these approaches is that fetal growth can be assessed only after delivery, and they do not allow different patterns of development to be examined throughout pregnancy. Thus, growth-retarded fetuses and healthy but constitutionally small ones may have the same birth weight (Gardosi 2004). In addition, it has been suggested that birth weight poorly reflects fetal growth during the first two trimesters of pregnancy (Gardosi 2004). Finally, assessment of birth size does not fully capture the time during gestation in which fetal growth failures begin or the onset of transient effects that may occur during intrauterine life. Therefore, the study of the effects of these contaminants on fetal growth using longitudinal ultrasound measurements may be useful to identify specific prenatal periods of vulnerability to OC exposure, and especially the age at which fetal growth failure may begin.

Within the Spanish INMA (INfancia y Medio Ambiente; Childhood and Environment) Project, we aimed to examine the relationship between maternal and cord concentrations of 4,4´-dichlorodiphenyldichloroethylene (4,4´-DDE), hexachlorobenzene (HCB), and three polychlorinated biphenyls (PCBs) (congeners 138, 153, and 180) and fetal growth using serial ultrasound measurements at 12, 20, and 34 weeks of pregnancy and taking into account the individual growth potential of the fetus.

## Material and Methods

*Study design and population*. The INMA Project is a multicenter population-based mother–child cohort study established in different areas of Spain following a common protocol (Guxens et al. 2012). This study included the cohorts of Asturias, Gipuzkoa, Sabadell, and Valencia. The hospital ethics committees of each region approved the research protocol.

A total of 2,644 eligible women (≥ 16 years, singleton pregnancy, enrollment at 10–13 weeks of gestation, nonassisted conception, delivery scheduled at the reference hospital, and no communication impairment) were recruited in the first trimester of pregnancy and gave their written informed consent before inclusion (2003–2008). After we excluded the women who withdrew from the study, were lost to follow-up, or had induced or spontaneous abortions or fetal deaths, 2,506 (95%) women were followed up to delivery (May 2004–August 2008). In the present study, the sample size was 2,407 mothers and their newborns (Table 1) with at least two valid ultrasounds and OC determinations in maternal (*n* = 2,369) and/or cord (*n* = 1,140) serum. The maternal and newborn characteristics of this study sample were comparable to those of the rest of the cohort (data not shown).

**Table 1:** Study population: the INMA Project, 2003–2008 (Spain) (*n* = 2,407).

Variable	Mean ± SD or *n* (%)
Maternal characteristics
Age (years)	31 ± 4.3
Height (cm)	163 ± 6.2
BMI^*a*^ (kg/m^2^)	24 ± 4.4
Recommended GWG^*b*^	890 (38)
Born in Spain	2,209 (92)
Primary studies	588 (25)
Working in pregnancy	2,008 (83)
Lowest social class	1,045 (43)
Rural residence	141 (6)
Primiparous	1,347 (56)
Smoking^*c*^	739 (32)
Passive smoking	1,452 (62)
Alcohol intake	299 (13)
Paternal characteristics
Height (cm)	176 ± 7.0
BMI (kg/m^2^)	26 ± 3.4
Child characteristics
Sex (male)	1,245 (52)
Abbreviations: BMI; body mass index; GWG; gestational weight gain. ^***a***^Prepregnancy BMI. ^***b***^Recommended GWG during the 2nd and 3rd trimester according to the prepregnancy BMI: 0.44–0.58, 0.35–0.50, 0.23–0.33, and 0.17–0.27 kg/week for underweight, normal, overweight, and obese women, respectively (Rasmussen et al. 2009). ^***c***^At week 12 of pregnancy.	

*OC exposure assessment*. We measured OC concentrations in maternal serum samples of the four cohorts and in umbilical cord serum samples of three cohorts (Asturias, Gipuzkoa, and Valencia). Samples collected in Gipuzkoa and Sabadell were analyzed at the Basque Government’s Public Health Laboratory in San Sebastian [limit of detection (LOD) of 0.071 ng/mL for all the OCs], and samples from Asturias and Valencia were analyzed at the Barcelona Institute of Environmental Assessment and Water Research (LODs of OCs between 0.010 and 0.035 ng/mL), as previously described (Goñi et al. 2007; Vizcaino et al. 2010). OC concentrations were determined by gas chromatography with electron capture detection and confirmation by gas chromatography coupled to a mass spectrometer detector. Both laboratories complied with the Arctic Monitoring and Assessment Program for persistent organic pollutants in human serum (Centre de Toxicologie, Institut National de Santé Publique du Québec). In this study, we present the results of those OCs with a detection frequency > 80% (4,4´-DDE, HCB, and PCBs 138, 153, and 180). Values below the LOD were replaced by values within the range [0, LOD] based on a multiple imputation procedure (see “Statistical analysis”).

We determined total cholesterol and triglycerides by enzymatic techniques, and calculated total serum lipid concentrations (Phillips et al. 1989). Means (± SDs) of total lipid contents in maternal and cord serum were 6.02 ± 1.10 and 2.53 ± 0.67 mg/mL, respectively.

*Fetal ultrasonography*. Ultrasound examinations were routinely scheduled for gestational weeks 12, 20, and 34, and were performed by obstetricians who specialized in conducting this type of examination at the respective hospitals. We also had access to the records of any other ultrasound scan performed on the women during pregnancy, which allowed us to obtain 2–8 valid ultrasound measurements per subject between the 7th and 42nd weeks of gestation. Of 2,478 women providing ultrasound data, 164 (6.6%) had two examinations, 2,035 (82.1%) had three, 235 (9.5%) had four, and 44 (1.8%) had five or more. Thus, we used a total of 7,602 scans to build longitudinal growth curves for fetal parameters (Iñiguez et al. 2013).

Gestational age was based on the self-reported date of the last menstrual period, but it was estimated using an early crown–rump length measurement if the self-reported and estimated dates differed by ≥ 7 days (Westerway et al. 2000). We removed women for whom this difference exceeded 3 weeks from the study to avoid possible bias (*n* = 18).

Fetal measures were abdominal circumference (AC), biparietal diameter (BPD), and femur length (FL). We also eliminated data outside the range of the mean ± 4 SDs for each gestational age to avoid the influence of extreme values (*n* = 5, 8, and 8 for AC, FL, and BPD, respectively). Additionally, we calculated estimated fetal weight (EFW) using the Hadlock algorithm (Hadlock et al. 1985). We used linear mixed models (Pinheiro and Bates 2000) separately in each cohort to obtain longitudinal growth curves for the four fetal parameters. Our aim was to discriminate between small fetuses (related to the size of the general population) and reduced growth (related with the characteristics of the fetus itself) (Mamelle et al. 2001). Therefore, we adjusted models for those available covariates related to the constitutional potential of the fetus (Mamelle et al. 2001). Fetal growth modeling is briefly described below and in greater detail in the Supplemental Material, “Detailed modeling procedure of mixed-effect models and growth curves,” Table S1, and Figures S1–S4.

The full model parameterization for each fetal parameter in each cohort is

*Y*^^(λ)^^*__ij__* = *X_ij_*β + *Z_ij_b_i_* + ε*_ij_*, [1]

where

*Y*^^(λ)^^*_ij_* is a Box-Cox transformation of the fetal parameter value *Y_ij_* in the *i*th fetus at time *T_j_*, suggested by Gurrin et al. (2001) and Royston and Altman (1994), in order to obtain normality and linearity;
*X_ij_* = [1, p(*T_ij_*), *C^1^_i_*,…,*C^P^_i_*, *T_ij_* × *C^1^_i_*,…,*T_ij_* × *C^P^_i_* ] is the fixed-effects regressor matrix for the *i*th fetus at time *T_j_*. p(*T_ij_*) represents a polynomial of entire order until 3 in *T_j_* or a low-order fractional polynomial, described by Royston and Altman (1994). (*C^1^_i_*,…,*C^P^_i_*) is the subset of the covariates considered for the *i*th fetus: maternal and paternal height, maternal and paternal weight or body mass index (BMI), maternal age, parity, country of origin, and fetal sex. Finally, (*T_ij_* × *C^1^_i_*,…, *T_ij_* × *C^P^_i_*) represent the interactions of each covariate with the time at measurement;
β is the vector of fixed coefficients to be estimated;
*Z_ij_* = [1, *T_ij_*] is the random-effects regressor matrix for the *i*th fetus at time *T_j_*;
*b_i_* is the vector of random effects that is estimated for each fetus, and whose distribution across the fetal population is assumed to be bivariate normal, *b_i_* = (*b_0i_*, *b_1i_*) ∝ *N* (0, *D*);
ε_ij_ is the within-subject error, ε_i_ = (ε_i1_,…,ε_iN_) ∝ *N* (0, σ^2^Λ_i_).

In our models the standard assumption of Λ_i_ = I was relaxed in order to allow for *a*) heteroscedasticity, and *b*) autocorrelation modeling of within-subject errors. This was performed in the following way:

Λ_i_ (*j,j*) = g(*T_ij_*, *C_i_*, *M_i_*, δ) [2]

Λ_i_ (*j,k*) = f(*d_jk_*, φ), [3]

where

g(x) is a function, assigning different variances to each value x, according to a parameter δ. Time (*T*), biological covariates (*C*), and a series of dichotomous variables (*M^1^_i_*,…,*M^^Q^^_i_*) tagging pregnancies with at least two consecutive ultrasounds performed too close together in time were tested as variables explaining heteroscedasticity. *M^j^* were often included in the models;
f(x) is a function modeling the auto-correlation structure within subjects. Different models of decays with distance were tested but in our models an exponential variogram was always selected. This model represents an exponential decay in the correlation between observations with the difference in time between them, that is, f (*d_jk_*,φ) = 1 – exp(*d_jk_*/φ). d_jk_ = |*T_ij_* – *T_ik_*| and φ parameter to be estimated.

The selection criterion to select the covariates included in *X*, *Z* as well as explaining heteroscedasticity for within-subject errors, was *p* < 0.05 (conditional *F*-test for fixed effects; *LR*-test for random effects).

Fetal growth curves provided predictions for weeks 12, 20, and 34, and were used to calculate unconditional and conditional SD:


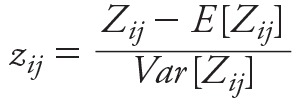
[4]


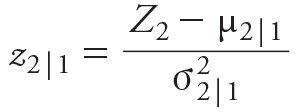
, _[5]_

where


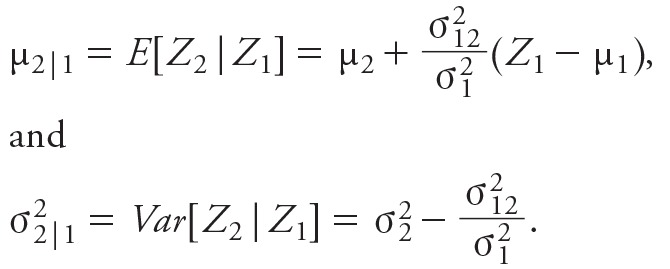
[6]

Unconditional scores [Equation 4] describe the size of a fetus and were calculated at 12, 20, and 34 weeks of gestation. Conditional SD scores [Equation 5]—that is, the standardization of the response at time T_2_, according to the observed value at time T_1_—evaluate the growth in the interval T_1_–T_2_, and were calculated for the intervals 12–20 and 20–34 weeks of gestation.

*Other covariates*. During the first and third trimesters of pregnancy, women completed two detailed in-person questionnaires on anthropometric, sociodemographic, and lifestyle characteristics, as well as two semi-quantitative food frequency questionnaires, as further described elsewhere (Llop et al. 2010). We considered the following maternal variables: age (years); height (centimeters); prepregnancy BMI (kilograms per meter squared); weekly gestational weight gain (GWG) from week 12 to delivery (low, recommended, and high) in accordance with the recommendations of the Institute of Medicine (Rasmussen et al. 2009); country of birth (Spain, Latin America, and other); area of residence (rural and urban); education (up to primary, secondary, and university); employment during pregnancy (yes and no); socioeconomic status according to the most privileged occupation of the mother or father during pregnancy [International Standard Classification of Occupations class I: managerial jobs, senior technical staff, and commercial managers; class II: skilled nonmanual workers; and class III: manual workers (Domingo-Salvany et al. 2000)]; parity (0 and ≥ 1 births); tobacco consumption at week 12 of pregnancy (yes and no); passive smoking at home, workplace, or leisure areas/restaurants (yes and no); season of last menstrual period; and intake of vegetables (grams/day), fruit (grams/day), seafood (including three variables: lean fish, oily fish, and other seafood in grams/day); total energy (kilocalories/day); and beverages containing alcohol (yes and no). We also considered paternal height (centimeters) and BMI (kilograms per meter squared), and sex of the fetus.

*Statistical analysis*. For descriptive purposes, we present numbers and percentages for categorical variables, and means and SDs for continuous variables. Percentiles (P) 25, P50, and P75 are presented for OCs. We evaluated placental transfer by calculating P25, P50, and P75 of the ratios of maternal and umbilical cord OC concentrations, and Pearson partial correlations between the compounds (log_2_-transformed) adjusted by cohort. We also used Pearson correlations adjusted by cohort to describe pairwise relationships between log_2_(OC) measured in the same matrix. Contaminant concentrations are expressed as wet-weight concentrations (nanograms per milliliter) and in nanograms per gram lipid for descriptive analyses.

We conducted multiple linear regression analyses to analyze the association between maternal and cord serum OC concentrations and SD scores of fetal growth measurements at weeks 0–12, 12–20, 20–34, and size at 34 weeks of gestation. We used wet-weight OC concentrations with adjustment for maternal or cord lipid levels as a separate term to minimize potential biases due to lipid standardization (Schisterman et al. 2005), after log_2_-transformation of OC [log_2_(OC)] and lipid concentrations to account for right skewed distributions. To identify predictor and confounder variables, we conducted linear regression analyses to determine covariates associated with fetal growth outcomes and Tobit regression to study the relationship between covariates and log_2_(OC) concentrations, accounting for the left-censoring of the data at the LOD (Lubin et al. 2004). The adjustment for covariates was performed in line with the following procedure. First, we included in basal models any variable associated with each outcome in bivariate analyses at a significance level of *p* < 0.20. Then, we sequentially excluded variables not related at *p* < 0.10 using the *F*-test and following a backward procedure. Later, we also added any potential confounder (included in the section of covariates) of OCs to the models if the OC coefficient changed by > 10% when it was added. Moreover, we included in all the models, regardless of their statistical significance, the following variables: cohort, total serum lipid levels, maternal age, maternal BMI, and maternal country of birth, because these four latter were strongly associated with OCs in previous studies (Ibarluzea et al. 2011; Llop et al. 2010; Vizcaino et al. 2010). GWG was also included in some models (Govarts et al. 2014; Verner et al. 2013; Vizcaino et al. 2014b). Specifically, GWG was included in models of cord OCs and outcomes measured from week 12 onward because GWG was calculated from week 12 to delivery. We assessed the normality and homoscedasticity of regression residuals, excluding extreme outliers (studentized residuals ≥ 4) or highly influential observations (Cook’s distance > 0.5) from the main analyses.

Final models were stratified by cohort to account for the possible heterogeneity of the association between exposure and response variables. Final coefficients and their 95% confidence intervals (CIs) were estimated using meta-analysis. Heterogeneity was quantified with the *I*^2^ statistic (Higgins et al. 2003) and the random-effects model was used when levels were > 50%. Parameter estimates were expressed as the percentage of change in the outcome with respect to the mean, and its 95% CI associated with a doubling in the OC concentrations.

We used multiple imputation with chained equations (Sterne et al. 2009) to deal with missing values in covariates and with values < LOD in exposure variables [avoiding the fixed imputation of LOD/2 by assuming a log-normal distribution of OCs and conditioning the imputation to the range (0, LOD)]. We generated a total of 50 complete data sets by using the *mice* package for R (van Buuren and Groothuis-Oudshoorn 2011), and estimates on each data set were combined using Rubin’s rules for multiple imputation (Little and Rubin 2002). To impute OC values < LOD, we defined an additional function for bootstrap multiple imputation of interval censored variables (Lubin et al. 2004). Information on the multiple imputation procedure is available in the Supplemental Material, “Details on multiple imputation (MI) modeling,” and Table S2. All results in the present work (including the description of OC concentrations, the Pearson correlation analyses, and the final cohort-specific adjusted models) were based on pooled estimates from a multiple imputed data set.

We carried out different sensitivity analyses to evaluate the robustness of the results. First, we compared models using multiple imputation (the main analysis of our study) with the complete case analysis (models restricted to subjects with complete data in covariates and replacing OC values < LOD with LOD/2 value). In models of multiple imputation, we conducted a multi-pollutant analysis that simultaneously included the OCs showing an association with fetal growth in the present analysis. We also ran an analysis excluding GWG. Finally, we investigated differences by sex by including the interaction of this variable with the contaminants in the main analyses.

We used the statistical software R.3.1.1 (R Core Team 2014). Where associations are referred to as statistically or marginally significant associations, this implies a *p*-value < 0.05 or < 0.1, respectively.

## Results

*Study population characteristics*. Table 1 shows the characteristics of the study population. Mean maternal age was 31 years (range, 16–43 years); 56% of the mothers were primiparous, and 32% were smokers at the beginning of pregnancy.

*OC concentrations*. Wet-weight concentrations of OCs in maternal and cord serum samples are shown in Table 2. We used individual total lipid values to calculate OC concentrations on a lipid content basis. Maternal medians for 4,4´-DDE, HCB, as well as for PCBs 138, 153, and 180, were 141, 50, 29, 48, and 34 ng/g lipid, respectively. Respective cord medians were 154, 61, 34, 48, and 34 ng/g lipid (data not shown).

**Table 2:** Percentage ≥ LOD and median (P25, P75) of OCs (ng/mL): the INMA Project, 2003–2008 (Spain).

Variable	4,4´-DDE	HCB	PCB-138	PCB-153	PCB-180
Maternal serum
Overall % ≥ LOD	99.2	93.2	90.9	96.2	93.6
Overall (*n* = 2,369)	0.83 (0.49, 1.56)	0.29 (0.16, 0.51)	0.17 (0.11, 0.25)	0.28 (0.19, 0.40)	0.20 (0.13, 0.30)
Asturias (*n* = 450)	1.39 (0.80, 2.41)	0.37 (0.22, 0.58)	0.20 (0.14, 0.29)	0.34 (0.24, 0.45)	0.24 (0.16, 0.34)
Gipuzkoa (*n* = 596)	0.54 (0.35, 0.84)	0.20 (0.12, 0.32)	0.18 (0.13, 0.26)	0.30 (0.21, 0.43)	0.22 (0.14, 0.34)
Sabadell (*n* = 594)	0.71 (0.43, 1.16)	0.23 (0.13, 0.38)	0.11 (0.07, 0.16)	0.20 (0.14, 0.28)	0.14 (0.09, 0.20)
Valencia (*n* = 729)	1.09 (0.64, 1.90)	0.43 (0.21, 0.69)	0.20 (0.13, 0.28)	0.30 (0.21, 0.41)	0.22 (0.15, 0.31)
Umbilical cord serum
Overall % ≥ LOD	98.3	87.7	81.8	91.6	86.3
Overall (*n* = 1,140)	0.38 (0.23, 0.67)	0.16 (0.09, 0.27)	0.08 (< LOD, 0.12)	0.12 (0.08, 0.17)	0.08 (< LOD, 0.12)
Asturias (*n* = 318)	0.47 (0.25, 0.85)	0.13 (0.09, 0.22)	0.08 (0.05, 0.12)	0.13 (0.09, 0.18)	0.07 (0.04, 0.10)
Gipuzkoa (*n* = 324)	0.24 (0.15, 0.37)	0.11 (< LOD, 0.17)	0.08 (< LOD, 0.12)	0.12 (0.08, 0.17)	0.10 (< LOD, 0.13)
Valencia (*n* = 498)	0.46 (0.30, 0.78)	0.22 (0.13, 0.36)	0.09 (0.05, 0.12)	0.11 (0.08, 0.16)	0.08 (0.05, 0.11)
Abbreviations: DDE, dichlorodiphenyldichloroethylene; HCB, hexachlorobenzene; LOD, limit of detection; OC, organochlorine compound; P, percentile; PCB, polychlorinated biphenyl.					

*Maternal and cord serum OC correlations and placental transfer of contaminants*. Three of the four participant cohorts (Asturias, Gipuzkoa, and Valencia) had information on both maternal and cord serum OC concentrations. Table 3 shows the Pearson correlation coefficients between maternal and cord log_2_(OC) concentrations expressed in ng/mL as well as in ng/g lipid (range, 0.38–0.77, *p* < 0.001 in all cases). Maternal serum concentrations (nanograms per milliliter) of DDE, HCB, and PCBs 138, 153, and 180 averaged 2.46, 2.11, 2.41, 2.55, and 2.79 times those of cord serum, respectively. Ratios were close to 1 when OC concentrations were lipid-adjusted (Table 3). In the Supplemental Material, Table S3 shows the Pearson’s correlations between OCs in the same matrix (*p* < 0.001 in all cases) being similar using the wet-weight and the lipid-adjusted concentrations. Correlations were lower between DDE and other OCs (ranges, 0.12–0.27 and 0.31–0.39 in maternal and cord serum, respectively), and higher between HCB and PCBs or between PCB congeners (ranges, 0.53–0.88 and 0.54–0.82 in maternal and cord serum, respectively).

**Table 3:** Concentration ratios and Pearson correlations of OCs in maternal and umbilical cord serum (*n* = 1,102): the INMA Project, 2003–2008 (Spain).

OC	Expressed in ng/mL		Expressed in ng/g lipid	
	Ratio^*a*^: C_m_/C_uc_ [median (P25, P75)]	Pearson^*b*^**coef	Ratio^*a*^: C_m_/C_uc_ [median (P25, P75)]	Pearson^*b*^**coef
4,4´-DDE	2.46 (1.88, 3.21)	0.77	1.04 (0.76, 1.46)	0.75
HCB	2.11 (1.49, 2.94)	0.57	0.89 (0.56, 1.36)	0.54
PCB-138	2.41 (1.69, 3.28)	0.41	1.01 (0.67, 1.47)	0.38
PCB-153	2.55 (1.93, 3.45)	0.49	1.11 (0.79, 1.52)	0.47
PCB-180	2.79 (1.96, 4.07)	0.56	1.19 (0.79, 1.79)	0.55
Abbreviations: coef, coefficient; DDE, dichlorodiphenyldichloroethylene; HCB, hexachlorobenzene; OC, organochlorine compound; P, percentile; PCB, polychlorinated biphenyl. Both maternal and cord concentrations were available for three out of the four cohorts (Asturias, Gipuzkoa, and Valencia). ^***a***^Ratio: C_m_/C_uc_: ratio of maternal/cord concentrations in raw scale. ^***b***^Pearson correlation coefficient between maternal and cord OC concentrations in log scale (adjusted by cohort). *p* < 0.001 in all the Pearson correlations.				

**Figure 1:**
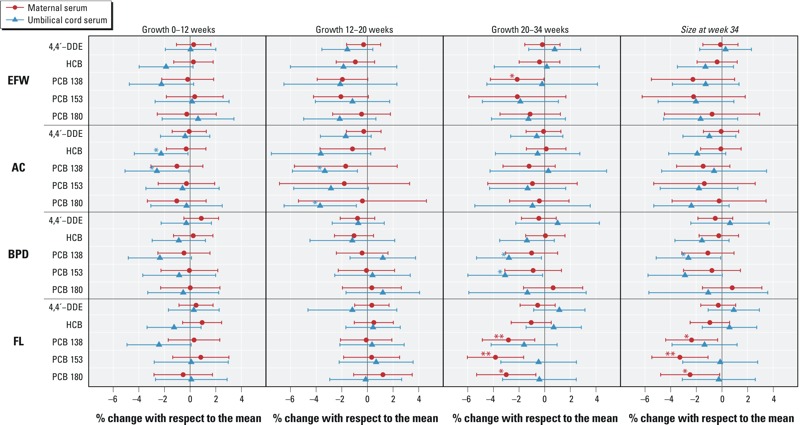
Associations between OC concentrations and fetal growth measurements: The INMA Project, 2003–2008 (Spain). Abbreviations: AC, abdominal circumference; BPD, biparietal diameter; DDE, dichlorodiphenyldichloroethylene; EFW, estimated fetal weight; FL, femur length; HCB, hexachlorobenzene; OC, organochlorine compound; PCB, polychlorinated biphenyl. Adjusted linear regression models between log_2_(OC) concentrations and fetal growth measurements. Meta-analysis of results from multiple imputation. Results expressed as percent change in fetal measurements associated with a doubling in OC concentrations. *p < 0.05. **p < 0.01.

*OC exposure and ultrasound measurements of fetal growth*. In Figure 1, the adjusted regression analyses are shown for the relationship between log_2_(OC) concentrations measured in either maternal or cord serum samples and SD scores of fetal growth or size (percent of change; 95% CI, for a doubling in OCs).

Between 0 and 12 weeks of pregnancy, there were not statistically significant associations between OC concentrations and fetal outcomes except in the case of cord HCB (–2.3%; 95% CI: –4.4, –0.2%) or cord PCB-138 (–2.6%; 95% CI: –5.1, –0.1%) and AC growth. Marginally significant inverse associations (*p* < 0.10) were also found between cord HCB and EFW and between cord PCB-138 and the rest of fetal outcomes.

For the period between 12 and 20 weeks of gestation, we did not find any statistically significant associations between OC concentrations and growth of BPD or FL. Regarding AC growth, patterns were similar for maternal and cord serum (Figure 1), but only statistically significant for cord PCB-138 (–3.3%; 95% CI: –5.8, –0.8%) and PCB-180 (–3.7%; 95% CI: –6.5, –0.9%). Marginally significant inverse associations were also found between AC and the rest of OCs measured in cord (4,4´-DDE, HCB, and PCB-153). Associations for EFW growth did not reach statistical significance but were marginal with maternal PCBs 138 and 153.

For the period between 20 and 34 weeks of pregnancy, negative associations were observed for all the fetal outcomes and PCBs measured in either maternal or cord serum. These associations were significant for maternal PCB-138 and EFW growth (–2.1%; 95% CI: –4.2, –0.1%), maternal PCBs and FL growth (PCB-138: –2.8%; 95% CI: –4.9, –0.8%; PCB-153: –3.8%; 95% CI: –6.0, –1.6%; and PCB-180: –3.0%; 95% CI: –5.3, –0.7%), and cord PCBs and BPD growth (PCB-138: –2.8%; 95% CI: –5.3, –0.3%; and PCB-153: –3.1%; 95% CI: –6.0, –0.2%).

Patterns of associations on growth parameters were coherent with those observed on size at 34 weeks, but only some associations were statistically significant (Figure 1). Negative associations between cord PCBs 138 and 153 and growth in BPD between 20 and 34 weeks of gestation were also apparent for the same exposures and BPD size at 34 weeks, though the association with PCB-153 was only marginally significant. All three PCBs measured in maternal serum were associated with significantly lower FL growth between 20 and 34 weeks of gestation and smaller FL size at 34 weeks. Although AC growth was significantly lower in association with cord HCB and PCB-138 at 0–12 weeks, and with cord PCBs 138 and 180 at 12–20 weeks, AC size at week 34 was not clearly associated with either exposure. No significant associations between DDE and fetal growth were observed during pregnancy.

**Figure 2:**
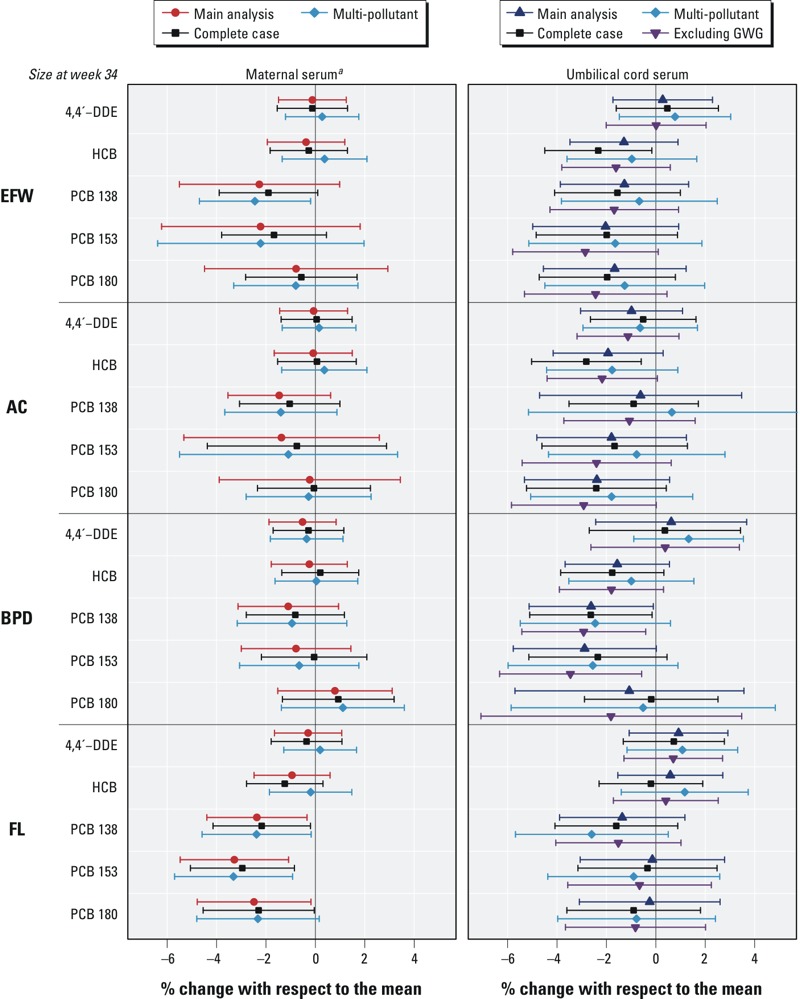
Sensitivity analysis of the association between OC concentrations and fetal size at 34 weeks of gestation: the INMA Project, 2003–2008 (Spain). Abbreviations: AC, abdominal circumference; BPD, biparietal diameter; DDE, dichlorodiphenyldichloroethylene; EFW, estimated fetal weight; FL, femur length; GWG, gestational weight gain; HCB, hexachlorobenzene; OC, organochlorine compound; PCB, polychlorinated biphenyl. Adjusted linear regression models between log_2_(OC) concentrations and fetal growth measurements. Meta-analysis of results from multiple imputation. Results expressed as percent change in fetal measurements associated with a doubling in OC concentrations. Main analysis: results from multiple imputation. Complete case: analysis excluding cases with missing values in covariates and fixed imputation of LOD/2 for OC values < LOD. Multi-pollutant: main analysis including the OCs showing an association with fetal growth in the present analysis, i.e., models of 4,4´-DDE were additionally adjusted for ∑PCBs and HCB, models of HCB were adjusted for ∑PCBs, and models of PCBs were adjusted for HCB. Excluding GWG: analysis excluding gestational weight gain. ***^a^***GWG was not included in models of maternal OCs and outcomes measured at week 12 because GWG was calculated from week 12 to delivery.

The estimates of the main analysis (i.e., multiple imputation) were similar to those of the complete case analysis (i.e., restricted to data with no missing information in covariates and OC values < LOD replaced with LOD/2 value), the multi-pollutant analysis (i.e., main analysis including other OCs), or the main analysis excluding GWG (Figure 2; see also Supplemental Material, Figures S5–S7). The main differences were found in the multi-pollutant analysis with wider CIs. There were no consistent interactions with sex (data not shown), and only two interactions were statistically significant: cord HCB and FL growth between 12 and 20 weeks of pregnancy (females: –1.9%; 95% CI: –4.9, 1.0%, and males: 2.3%; 95% CI: –0.4, 5.0%, *p*-interaction = 0.03) and maternal 4,4´-DDE and AC growth between 20 and 34 weeks of gestation (females: 1.5%; 95% CI: –0.6, 3.5%, and males: –1.2%; 95% CI: –2.9, 0.6%, *p*-interaction = 0.05).

## Discussion

To the best of our knowledge, this is the first study to consider specific fetal body segments that could be affected by exposure to OCs during different critical exposure windows. Increases in PCB concentrations were related to reductions in AC up to mid-pregnancy, and to decreases in fetal BPD and FL from gestational week 20 onward. We also found an inverse association between HCB and AC during the first trimester of pregnancy. The estimated mean difference in these fetal parameters ranged from –4% to –2% for a doubling in the OC concentrations. The magnitudes of the estimates in the multiple imputation analysis were similar to those of the complete case analysis, as well as those of the multiple imputation analysis after adding other OCs or excluding GWG. Clear results suggesting a differential effect between sexes were not found.

No associations between serum DDE concentrations and ultrasound measurements were found. No previous studies using fetal anthropometry measures are available for comparison with our results, but controversy exists about birth size. Although associations were reported in some studies (Lopez-Espinosa et al. 2011; Wolff et al. 2007), others found little or no evidence of associations with DDE exposure (Govarts et al. 2012; Sagiv et al. 2007).

Cord serum HCB concentrations were inversely associated with AC in early pregnancy. Although this fetal measure and birth weight are not directly comparable, a marginally significant decrease in birth weight associated to cord HCB concentrations was reported in newborns from the INMA-Valencia cohort (Lopez-Espinosa et al. 2011). Additionally, a non-statistically significant inverse association between birth weight and maternal HCB concentrations was reported in another previous INMA study (Basterrechea et al. 2014).

Some PCBs measured in cord serum were negatively associated with AC. Specifically, this was the case with PCB-138 up to the second trimester and PCB-180 at 12–20 weeks of pregnancy. Despite the limitations of comparability between fetal and birth outcomes, a systematic analysis of 20 epidemiological studies on PCBs reported insufficient evidence of an association with birth weight < 2,500 g (El Majidi et al. 2012). Conversely, an inverse linear exposure–response relationship between birth weight and cord PCB-153 was reported in a meta-analysis conducted in 12 European cohorts, which include the children in the present study (Casas et al. 2015; Govarts et al. 2012). In a second analysis controlling for GWG, the strength of the association was reduced, although a statistically significant reduction in birth weight was still observed (Govarts et al. 2014). In the present study, maternal serum PCB-138, -153, and -180 concentrations were associated with lower FL growth from 20 to 34 weeks and smaller FL size at 34 weeks. Cord serum PCB-138 and -153 concentrations were associated with lower BPD growth from 20 to 34 weeks and smaller BPD size at 34 weeks (with the latter significant only for PCB-138). A marginally significant reduction in birth length associated with cord PCB-153 concentrations was previously found in the INMA-Valencia cohort (Lopez-Espinosa et al. 2011). Conversely, associations with birth length or head circumference were not found in other studies (Sagiv et al. 2007; Wolff et al. 2007).

Although the biological mechanisms underlying the effects of OCs on fetal growth are not well established, these compounds can disrupt the endocrine system, which is involved in fetal development (Bourguignon and Parent 2010). Thus, thyroid hormones play an important role in somatic growth and in the differentiation and functioning of many tissues during development (Blazer et al. 2003), and some studies have suggested the existence of an association between altered thyroid levels during pregnancy and exposure to some OCs (Alvarez-Pedrerol et al. 2009; Lopez-Espinosa et al. 2009). OCs may also impede placental functions and contribute to fetal growth impairment. Thus, exposure to some OCs has been associated with placental vascular and trophoblastic lesions in animals studies (Bäcklin et al. 1998) and alterations of the placental transport of calcium and other nutrients in humans (Hamel et al. 2003; Tsuji et al. 2013) that are essential for fetal development.

Several shortcomings of the present study warrant cautious interpretation of the findings until more studies are available. Because multiple estimates were derived, results should be taken with caution: Some statistically significant associations could result from chance. The estimates of the coefficients and their confidence intervals should be taken as a global picture of the pattern of the relations between the variables involved in the study (Rothman 1990). Second, the criteria of inclusion may have imposed some selection and an underrepresentation of pregnant women with increased risk of adverse pregnancy outcomes. However, the aim of these commonly used criteria is to obtain a more homogeneous population and reduce the confounding potential. Another weakness is the possible selection bias between women included in or excluded from the present study, yet the differences in the main study variables observed between both groups were not significant. Although the OC concentrations were measured in two different laboratories with different LODs, both participated in the same monitoring and assessment program for persistent organic pollutants in human serum to verify their analytical results and to ensure the comparability of their data. In addition, random-effects meta-analysis was used to address heterogeneity resulting from the use of different laboratories and other factors that could differ among the cohorts.

One of the major strengths of this work with respect to previous studies on birth size is the repeated measurements of fetal anthropometry, which allowed us to study associations between OCs and growth in different stages of pregnancy. We accurately assessed fetal growth by means of a longitudinal analysis, adjusting for parental and fetal characteristics, to compare the expected versus real growth of each fetus. The use of individualized standards is expected to reduce misclassification by identifying constitutionally small babies and those with restricted growth (Gardosi 2004). Second, the use of repeated measurements of fetal anthropometry allowed us to study associations between these growth parameters and OCs in different stages of pregnancy, and thereby identify critical periods within gestation. Third, unlike most previous studies that have relied on a single blood measurement of exposure, we had information on OC exposure at the beginning of pregnancy and at delivery. Finally, other strengths of the present study are the large sample size, its prospective design, the low rate of participant dropout between recruitment and delivery, detailed information on many potential confounders from early pregnancy, and the use of multiple imputation to deal with undetected values in the exposure variables and missing values in the covariates (Sterne et al. 2009).

## Conclusions

PCB exposure may decrease fetal AC growth during the first two trimesters of pregnancy, and fetal growth of BPD and FL from mid-pregnancy onward. A transient association between HCB and AC in early pregnancy was also found. The reduction of these parameters ranged from –4% to –2% for a doubling in the OC levels. No statistically significant association between DDE and fetal growth was observed. Ultrasound measurements constitute a promising tool to examine how early prenatal OC exposure may affect fetal growth and more studies are needed.

## Supplemental Material

(4.3 MB) PDF
