# Supplementary material for: Organochlorine Compounds and Ultrasound Measurements of Fetal Growth in the INMA Cohort (Spain)
Source: Environ Health Perspect. 2015 Jun 9;124(1):157–63. doi: 10.1289/ehp.1408907 (PMC4710595; doi:10.1289/ehp.1408907)
Supplement: (4.3 MB) PDF [file ehp.1408907.s001.acco.pdf]

Environ Health Perspect
DOI: 10.1289/ehp.1408907

Note to Readers: *EHP* strives to ensure that all journal content is accessible to all readers. However, some figures and Supplemental Material published in *EHP* articles may not conform to 508 standards due to the complexity of the information being presented. If you need assistance accessing journal content, please contact ehp508@niehs.nih.gov. Our staff will work with you to assess and meet your accessibility needs within 3 working days.

Supplemental Material

Organochlorine Compounds and Ultrasound Measurements of Fetal Growth in the INMA Cohort (Spain)

Maria-Jose Lopez-Espinosa, Mario Murcia, Carmen Iñiguez, Esther Vizcaino, Olga Costa, Ana Fernández-Somoano, Mikel Basterrechea, Aitana Lertxundi, Mònica Guxens, Mireia Gascon, Fernando Goñi-Irigoyen, Joan O. Grimalt, Adonina Tardón, and Ferran Ballester

Table of Contents

Detailed modeling procedure of mixed-effect models and growth curves

Rationale

General model description

Conditional and unconditional centiles

Steps of the modeling procedure

Table S1. Summary of the models by cohort. The INMA Project, 2003-2008 (Spain).

Figure S1. Fetal growth curves for estimated fetal weight (EFW) in the four INMA-cohorts, 2003-2008 (Spain)

Figure S2. Fetal growth curves for abdominal circumference (AC) in the four INMA-cohorts, 2003-2008 (Spain).

Figure S3. Fetal growth curves for biparietal diameter (BPD) in the four INMA-cohorts, 2003-2008 (Spain)

Figure S4. Fetal growth curves for femur length (FL) in the four INMA-cohorts, 2003-2008 (Spain). There were not different FL curves by sex since sex did not enter in any model.

Details on the multiple imputation (MI) modeling

Imputation method

Software and statistical packages

Number of imputed datasets and iterations

Variables included in the imputation procedure

Heterogeneity in the imputation modeling

Criteria of inclusion

Diagnostics

Table S2. Number (%) of imputed values and regression model for each variable. The INMA Project, 2003-2008 (Spain).

Table S3. Pearson's correlations between OCs in maternal and umbilical cord serum. The INMA Project, 2003-2008 (Spain).

Figure S5. Sensitivity analyses of the associations between OC concentrations and fetal growth measurements between 0-12 weeks of gestation. The INMA Project, 2003-2008 (Spain).

Figure S6. Sensitivity analyses of the associations between OC concentrations and fetal growth measurements between 12-20 weeks of gestation. The INMA Project, 2003-2008 (Spain).

Figure S7. Sensitivity analyses of the associations between OC concentrations and fetal growth measurements between 20-34 weeks of gestation. The INMA Project, 2003-2008 (Spain).

Footnote of the Supplemental Material, Figures S5-S7

References

Detailed modeling procedure of mixed-effect models and growth curves

Rationale

The purpose of building fetal growth curves in the INMA-Project is to establish a relationship between the given fetal characteristics and gestational age in the INMA population, taking into account those non-pathological biological factors that may influence the growth potential of each fetus, and then to use these curves to estimate possible intrauterine restrictions of growth at several times within pregnancy.

Theoretically, considering the constitutional potential of each fetus should allow us to discriminate better between small fetuses (related to the size of the general population) and reduced growth (related with the characteristics of the fetus itself) (Mamelle et al. 2001).

General model description

The data for a single fetal parameter consist of vectors of observations:

$$\left\{\left(Y_{ij}; T_{ij}; C_i^p; M_i^q\right), \quad i = 1,\ldots,n;\ j = 1,\ldots,N_i;\ p = 1,\ldots,P; q = 1,\ldots,Q\right\},$$

where T_{ij} is the jth time-point in days for the ith fetus and Y_{ij} is the corresponding measurement. $\left(C_i^1,\ldots,C_i^P\right)$ are the paternal and fetal characteristics identified in the literature as possibly influencing fetal growth. $\left(M_i^1,\ldots,M_i^Q\right)$ are dichotomous variables tagging pregnancies with at least two consecutive ultrasounds performed too close together in time under different definitions of "too close". The response variable Y_{ij} was transformed searching for the linearity of the within-subject relationship with time. The transformation of the response, suggested in Gurrin et al. (2001) and Royston and Altman (1994), is a modification of the power transformation suggested by Box and Cox which takes: $Y_{ij}^{(\lambda)} = \begin{cases} Y_{ij}^{\lambda} & \lambda \neq 0 \\ \log(Y_{ij}) & \lambda = 0 \end{cases}$.

For the same purpose, we tested a polynomial of entire order until 3 in T_j or a low-order fractional polynomial, described by Royston and Altman (1994) in order to model the shape of response over time.

The full model is thus written:

$$Y_{ij}^{(\lambda)} = X_{ij}\beta + Z_{ij}b_i + \varepsilon_{ij}$$

where:

- $X_{ij} = \left[1,\ p(T_{ij}),\ C_i^1,...,C_i^P,\ T_{ij} \times C_i^1,...,T_{ij} \times C_i^P\right]$ and β is the corresponding vector of fixed coefficients to be estimated.

- $p(T_{ij})$ is a subset of the columns of $\left[T_{ij}, T_{ij}^2, T_{ij}^3\right]$ or an element of the class of fractional polynomial of degree 2: $\left[T_{ij}^{p1}, T_{ij}^{p2}\right]$, with $p1, p2 \in \left\{-2,-1,-\frac{1}{2},0,\frac{1}{2},1,2,3\right\}$ and with pi=0 corresponding to the logarithmic transformation.

- $\left(C_i^1,...,C_i^P\right)$ are the subset of the biological determinants considered: maternal and paternal height, maternal and paternal weight or body mass index (BMI), maternal age, parity, country of origin, and fetal sex. We checked whether they were reasonable under different metrics. $\left(T_{ij} \times C_i^1,...,T_{ij} \times C_i^P\right)$ are their interactions with the time at measurement.

- $Z_{ij} = \left[1, T_{ij}\right]$ represents the individual deviations from the mean of the fetal parameter for the population: constant deviations and linear change over gestation are allowed. b_i is the corresponding vector of random effects which is estimated for each fetus, and whose distribution across the fetal population is assumed to be bivariate normal: $b_i = (b_{0i},\ b_{1i}) \propto N(0, D)$. b_i is assumed to be independent among the subjects.

- ε_{ij} is the random variable representing the deviation in size at each time j on the ith fetus from the mean size. ε_i are called within-subject errors and are assumed to be bivariate normal: $\varepsilon_i = (\varepsilon_{i1},...,\varepsilon_{iN}) \propto N(0, \sigma^2\Lambda_i)$. The specification of the model additionally requires the independence of within-subjects residuals between subjects.

Commonly, although not necessarily, the independence of ε_{ij} within subjects (that is, Λ_i=I) is also specified, but in our case we used the extended model to allow for: 1) heteroscedasticity, and 2) autocorrelation of within-subject errors. This was performed in the following way:

1) $\sigma^2\Lambda_i(j,j) = var(\varepsilon_{ij}) = \sigma^2 \cdot g(T_{ij}, C_i, M_i, \delta)$,

where g is a function of at least one of the following variables: time, biological covariates, and

the dummies identifying subjects with an atypical sequence of ultrasound times:

$$M_i^q = \begin{cases} 0 & |T_{ij} - T_{ik}| > q, \ \forall \mathrm{j,k} \\ 1 & \textit{other} \ \text{case} \end{cases} \quad \mathrm{q} = 18, 21, 30 \ \text{days}$$

Several possibilities are implemented in R by default to be used as g functions. In our models, one of the $\mathrm{M}_\mathrm{i}^\mathrm{q}$ was commonly selected as influencing variance, in which case the g function consists in simply assigning different variances for each category. In all cases, the greater assigned variance matched the category of atypical mothers.

2) $\sigma^2 \Lambda_\mathrm{i}(\mathrm{j,k}) = \mathrm{cor}(\varepsilon_{\mathrm{ij}}, \varepsilon_{\mathrm{ik}}) = \sigma^2 \mathrm{f}(\mathrm{d}_{\mathrm{jk}}, \phi)$,

where f is a function which usually decreases with the distance between observations: $\mathrm{d}_{\mathrm{jk}} = |\mathrm{T}_{\mathrm{ij}} - \mathrm{T}_{\mathrm{ik}}|$, ϕ parameter to be estimated. Different functions are available in R to be used here as f, including well known from time-series or spatial data theory, are: AR, MA, ARMA, CAR, or exponential or Gaussian variograms. In our models, the most commonly selected function was the exponential variogram representing an exponential decay in the correlation between observations with the difference in time between them, that is, f $(\mathrm{d}_{\mathrm{jk}},\phi)$= 1-exp$(\mathrm{d}_{\mathrm{jk}}/\phi)$**.**

Conditional and unconditional centiles

The subsequent development and notation closely follow that of Royston (1995) and Gurrin et al. (2001) and further information may be found there. For each fetal dimension, once the corresponding linear mixed model was adjusted, the customized deviation of size in the ith fetus at time j, in relation to its potential, may be obtained in the usual way by employing the modeled mean and variance of the transformed response, Z=$\mathrm{Y}^{(\lambda)}$, at time j:

$$z_{ij} = \frac{Z_{ij} - E[Z_{ij}]}{Var[Z_{ij}]}$$

These are unconditional relative deviations, describing only a deviation in size, as any other information except the time and the characteristics of the fetus itself has been considered.

The linear mixed model assumes that the series of measurements within a given fetus have a multivariate normal distribution, hence implying that both marginal and conditional distributions of each pair of measurements Z_2 and Z_1 are univariate normal and the conditional distribution of

Z_2 given Z_1 is univariate normal with mean and variance: $\mu_{2|1} = E[Z_2 \mid Z_1] = \mu_2 + \frac{\sigma_{12}^2}{\sigma_1^2}(Z_1 - \mu_1)$,

$\sigma_{2|1}^2 = \mathrm{Var}[Z_2 \mid Z_1] = \sigma_2^2 - \frac{\sigma_{12}^2}{\sigma_1^2}$

The conditional deviation defined by:

$$z_{2|1} = \frac{Z_2 - \mu_{2|1}}{\sigma_{2|1}^2}$$

is the standardization of the transformed response at time $T_{2,}$ according to its conditional mean and variance at time T_2 given the observed value at time T_1.

That is, the status of the ith fetus at time T_1 is taken into consideration to update the mean and variance that should be used as a reference in T_2.

In our case, unconditional centiles were calculated for j=12, 20 and 34 weeks of gestation and conditional centiles were calculated for the intervals: 12–20, 12–34 and 20–34 weeks. Most women had ultrasound measurements at approximately 12, 20 and 34 weeks but not exactly at these points. Searching for the synchronization of outcomes, we calculated SD scores at a particular time, using the prediction at this particular time point conditioned to the nearest measure. That is, for example, if an ultrasound was performed at week 19, the SD score for week 20 was calculated in the standard way but using the prediction (from the modeled curve) of size at week 20 given the attained size at week 19 instead of the measured size at week 19. This procedure was used to prevent an increase in random error caused by the misalignment of measurements and by itself guarantees a complete data basis with SD close to 0 when there is a gap in the planned schedule of ultrasounds at weeks 12, 20 and 34.

Steps of the modeling procedure

For each fetal dimension in each cohort dataset:

1.) Estimation of λ for Box-Tidwell transformation of response: Searching for the normality in residuals of groups by a cubic polynomial of T. Functions: *aov* and *boxcox* (MASS library) (Gurrin et al. 2001).

2.) Selection of the best function to describe the change of parameters over time, that is, the specification of p(T). Functions: *glm* and *mfp* (mfp library). Selection criterion: minimum AIC.

3.) Introduction of covariates at intercept: applied on all but M_i. Method: forward. Function: gls (nlme library), in close connection with GEE (Pinheiro and Bates 2000), ML estimation. Selection criterion: LR test (p-value<0.05).

4.) Introduction of covariates interacting with time: as in 3.) and re-evaluation of covariates at intercept.

5.) Specification of correlation structure for within-subject errors. Covariates considered: T. Possible structures: CAR and variograms: exponential, gaussian, spherical, linear, rational squared (Pinheiro and Bates 2000). Selection criteria: minimum AIC over those structures which were significant (LR test; p-value<0.05) and presented no over-fitting (pACF of normalized residuals inspection). Again, re-evaluation of terms currently in the model.

6.) Specification of variance structure for within-subject errors. Covariates considered: T, C, M. Possible structures: varPower (for continuous covariates), varIdent (for categorical covariates) or a combination (Pinheiro and Bates 2000). Selection criterion: minimum AIC over those structures which were significant (LR test; p-value<0.05). Again, re-evaluation of terms actually in the model.

7.) Random-effects incorporation: tested if only at intercept, only at slope or in both terms. Functions: *gls* (nlme library), *lme* (nlme library). Selection criteria: Conditional F-test comparing with the full gls model re-fitted by REML (p-value<0.05) and no over-fitting given by the previously included correlation structure.

8.) Diagnosis: Normalized residuals should be N(0,I), random effects should be N(0,D), and independent among subjects. If necessary, go back to 2.).

9.) Prediction of aligned estimates to be used as observations at weeks 12, 20 and 34, and to obtain SD scores as previously described.

Table S1. Summary of the models by cohort. The INMA Project, 2003-2008 (Spain).

FW	Asturias	Gipuzkoa	Sabadell	Valencia
λ	log	log	0.06	log
P(T) order	3	2	3	2
Maternal age		x	x	x
Maternal height		x	x	
Paternal height	x			x
Maternal weight/BMI	x	x		x
Paternal weight/BMI			x	
Parity				
Country of origen		x	x	x
Sex	x	x	x	x
Variance structure	M^{21}, parity	parity, T	M^{30}	M^{30}

AC	Asturias	Gipuzkoa	Sabadell	Valencia
λ	0.34	0.45	0.59	0.44
P(T) order	3	3	3	3
Maternal age		x	x	x
Maternal height			x	
Paternal height	x			x
Maternal weight/BMI	x	x	x	x
Paternal weight/BMI		x	x	
Parity				
Country of origen		x	x	
Sex	x	x	x	x
Variance structure	M^{30}, T		parity	M^{30}

BPD	Asturias	Gipuzkoa	Sabadell	Valencia
λ	0.64	0.62	0.78	0.67
P(T) order	2	3	3	2
Maternal age		x		x
Maternal height	x	x	x	
Paternal height				
Maternal weight/BMI		x		x
Paternal weight/BMI	x	x	x	x
Parity	x			x
Country of origen			x	x
Sex	x	x	x	x
Variance structure	M^{18}	country, parity		M^{21}

FL	Asturias	Gipuzkoa	Sabadell	Valencia
λ	0.69	0.74	0.80	0.79
P(T) order	3	3	3	3
Maternal age		x	x	x
Maternal height	x	x	x	x
Paternal height	x	x		x
Maternal weight/BMI	x			x
Paternal weight/BMI			x	
Parity				x
Country of origen		x	x	
Sex				
Variance structure	M^{30}, parity	M^{30}, T	M^{21}, T	T

Correlation structure was an exponential variogram in all cases and random effects were never incorporated.

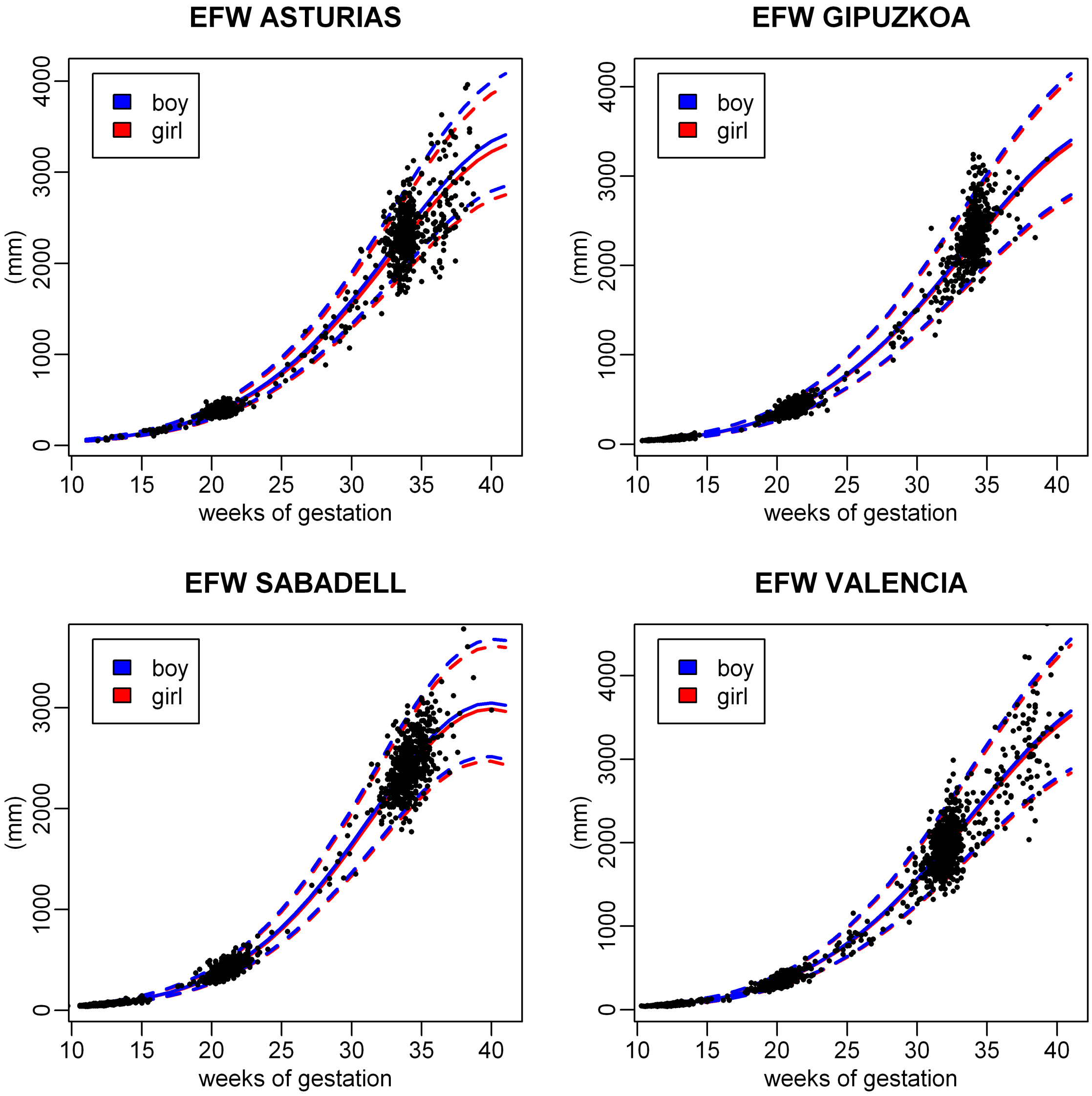

Figure S1. Fetal growth curves for estimated fetal weight (EFW) in the four INMA-cohorts, 2003-2008 (Spain)

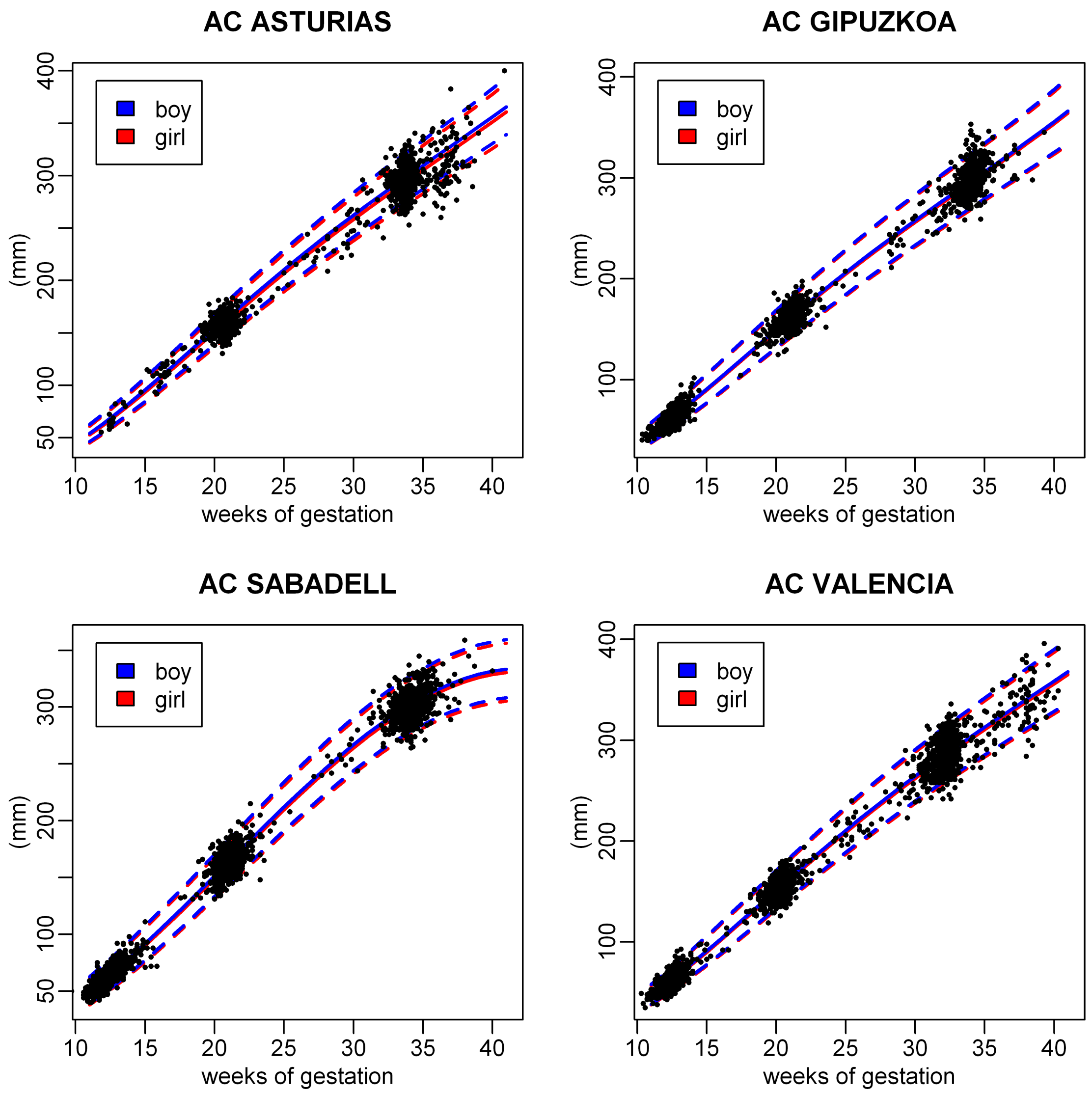

Figure S2. Fetal growth curves for abdominal circumference (AC) in the four INMA-cohorts, 2003-2008 (Spain).

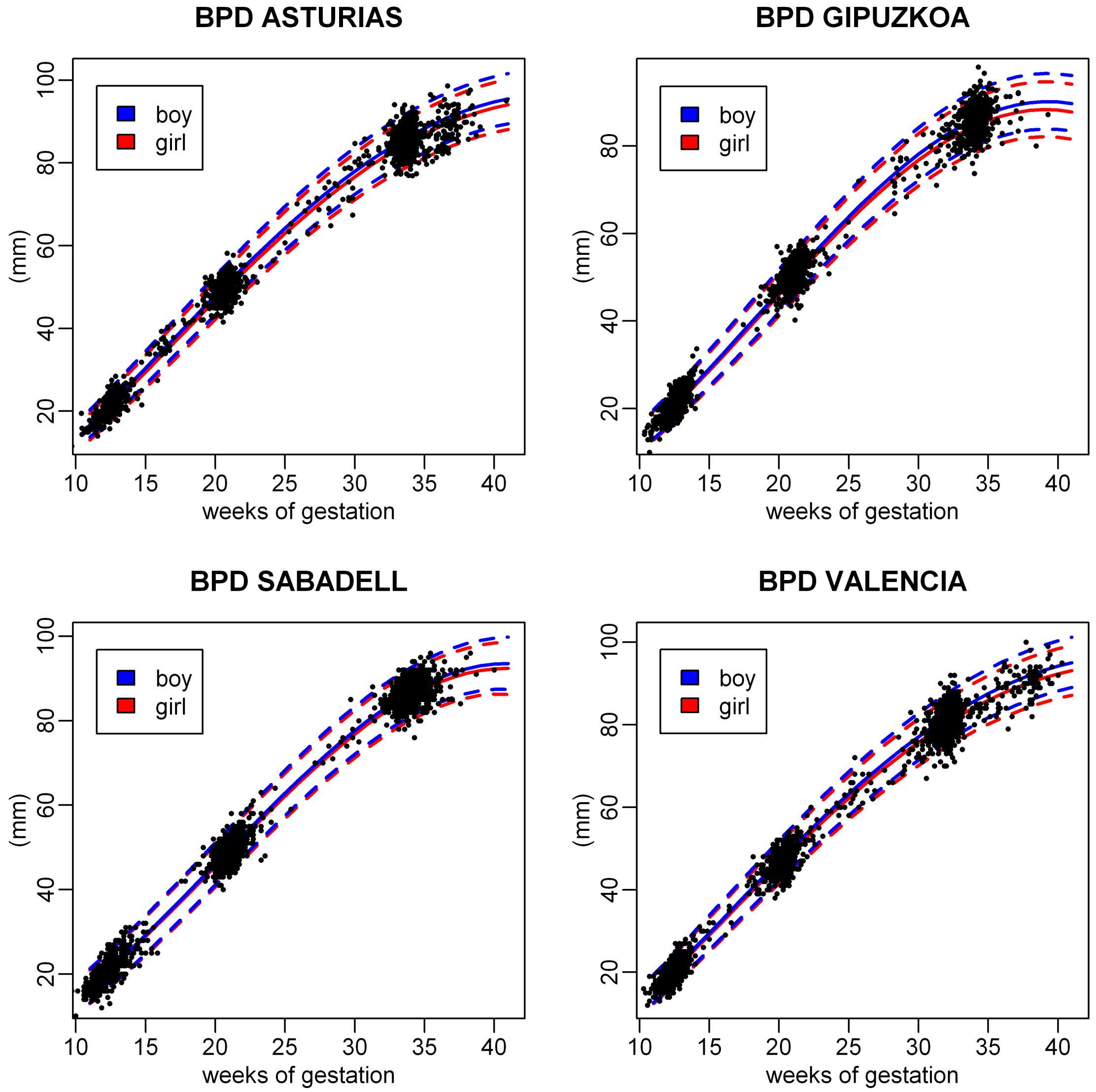

Figure S3. Fetal growth curves for biparietal diameter (BPD) in the four INMA-cohorts, 2003-2008 (Spain)

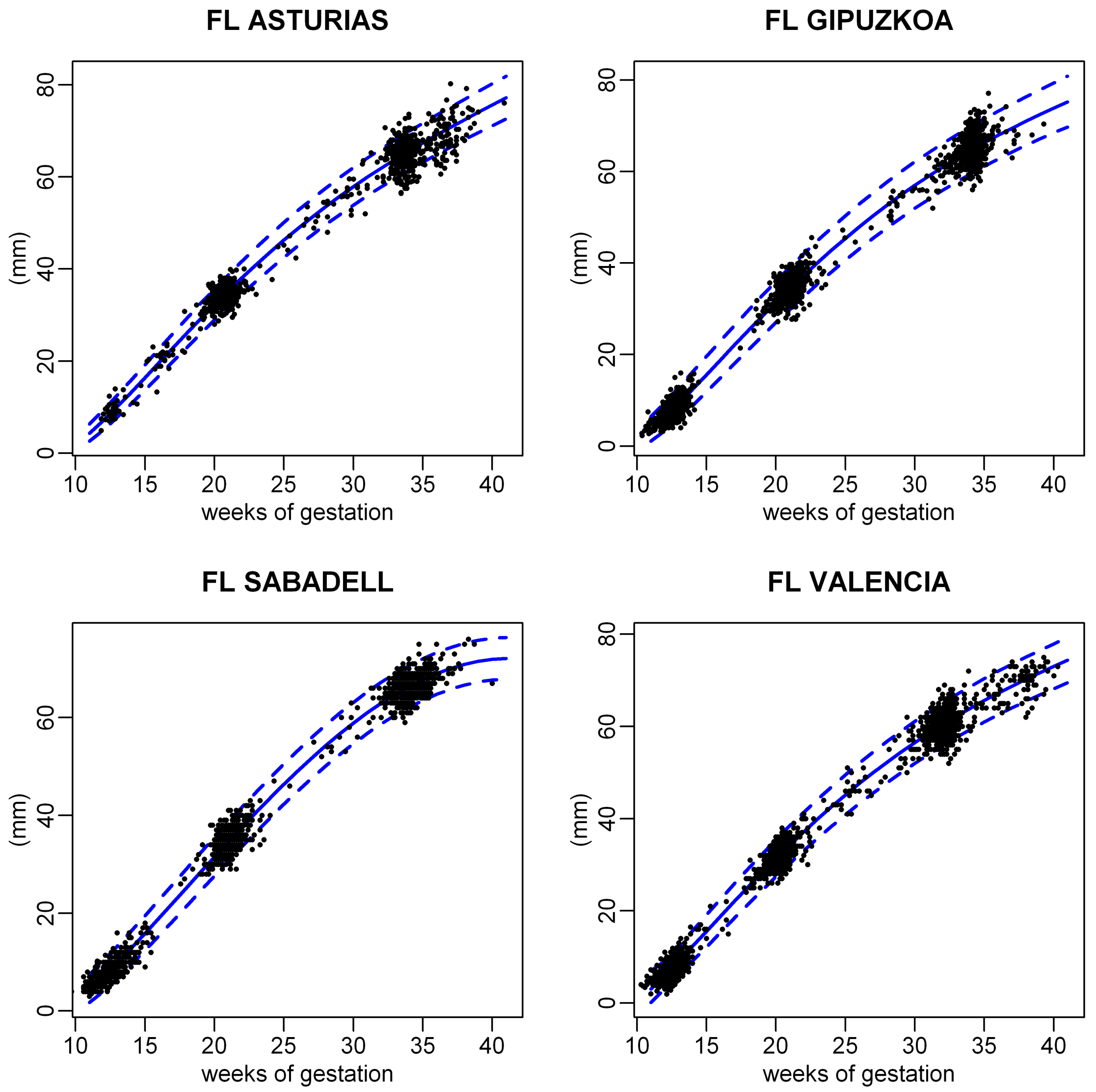

Figure S4. Fetal growth curves for femur length (FL) in the four INMA-cohorts, 2003-2008 (Spain). There were not different FL curves by sex since sex did not enter in any model.

Details on the multiple imputation (MI) modeling

Imputation method: fully conditional specification or multivariate imputation by chained equations (Horton and Kleinman 2007;Van Bauren and Groothuis-Oudshoorn 2011).

Software and statistical packages: R.3.1.1 (R Core Team 2014), *mice* package (Van Bauren and Groothuis-Oudshoorn 2011). An additional function was defined for bootstrap multiple imputation of censored variables (Lubin et al. 2004).

Number of imputed datasets and iterations: we imputed 50 datasets in order to diminish simulation error, each one with 20 cycles.

Variables included in the imputation procedure: outcome and exposure variables, covariates and potential confounders, and other variables not included in the main analyses but possibly related with variables which have a moderate number of missing/censored values (Supplemental Material, Table S2).

Heterogeneity in the imputation modeling: statistical interactions were not included in the imputation models; however, we performed multiple imputation stratified by cohort (Asturias, Gipuzkoa, Sabadell, and Valencia), since our final results were obtained using meta-analyses to account for possible heterogeneity (Graham 2009).

Criteria of inclusion: variables were included in the models for MI based on their prediction ability (correlation) and their relation to the non-response, excluding variables with too many missing values within the subgroup of incomplete cases (proportion of usable cases) (Van Bauren and Groothuis-Oudshoorn 2011). In order to avoid bias, outcome variables were used to impute exposure variables and vice versa (Van Bauren and Groothuis-Oudshoorn 2011;von Hippel 2007).

Diagnostics: Convergence was assessed by plotting parameters (mean and standard deviation in each imputed dataset) against iteration number. Imputations of missing and censored values were checked graphically and compared with observed data. Results from multiple imputation *versus* complete case analysis were also shown.

Table S2. Number (%) of imputed values and regression model for each variable. The INMA Project, 2003-2008 (Spain).

N: 2407 cases with ultrasound measurements and OCs in maternal and/or cord serum.

Outcome variables[a]	Imputed		Missing		Censored		Method
	n	(%)	*n*	(%)	*n*	(%)	
EFW: growth between 0–12 weeks	19	(0.8)	19[a]	(0.8)	0	(0.0)	linear regression
EFW: growth between 12–20 weeks	20	(0.8)	20[a]	(0.8)	0	(0.0)	linear regression
EFW: growth between 20–34 weeks	15	(0.6)	15[a]	(0.6)	0	(0.0)	linear regression
EFW: size at week 34	16	(0.7)	16[a]	(0.7)	0	(0.0)	linear regression
AC: growth between 0–12 weeks	11	(0.5)	11[a]	(0.5)	0	(0.0)	linear regression
AC: growth between 12–20 weeks	9	(0.4)	9[a]	(0.4)	0	(0.0)	linear regression
AC: growth between 20–34 weeks	8	(0.3)	8[a]	(0.3)	0	(0.0)	linear regression
AC: size at week 34	6	(0.2)	6[a]	(0.2)	0	(0.0)	linear regression
BPD: growth between 0–12 weeks	5	(0.2)	5[a]	(0.2)	0	(0.0)	linear regression
BPD: growth between 12–20 weeks	6	(0.2)	6[a]	(0.2)	0	(0.0)	linear regression
BPD: growth between 20–34 weeks	4	(0.2)	4[a]	(0.2)	0	(0.0)	linear regression
BPD: size at week 34	4	(0.2)	4[a]	(0.2)	0	(0.0)	linear regression
FL: growth between 0–12 weeks	10	(0.4)	10[a]	(0.4)	0	(0.0)	linear regression
FL: growth between 12–20 weeks	11	(0.5)	11[a]	(0.5)	0	(0.0)	linear regression
FL: growth between 20–34 weeks	7	(0.3)	7[a]	(0.3)	0	(0.0)	linear regression
FL: size at week 34	7	(0.3)	7[a]	(0.3)	0	(0.0)	linear regression
**Exposure variables**							
log(maternal 4,4′-DDE)	57	(2.4)	38[a]	(1.6)	19	(0.8)	censored linear regression
log(maternal HCB)	200	(8.3)	38[a]	(1.6)	162	(6.7)	censored linear regression
log(maternal PCB 138)	254	(10.6)	39[a]	(1.6)	215	(8.9)	censored linear regression
log(maternal PCB 153)	129	(5.4)	39[a]	(1.6)	90	(3.7)	censored linear regression
log(maternal PCB 180)	189	(7.9)	38[a]	(1.6)	151	(6.3)	censored linear regression
log(maternal ΣPCBs)	287	(11.9)	40[a]	(1.7)	247	(10.3)	passive imputation
log(cord 4,4′-DDE)	1287	(53.5)	1267[a,b]	(52.6)	20	(0.8)	censored linear regression
log(cord HCB)	1407	(58.5)	1267[a,b]	(52.6)	140	(5.8)	censored linear regression
log(cord PCB 138)	1474	(61.2)	1267[a,b]	(52.6)	207	(8.6)	censored linear regression
log(cord PCB 153)	1363	(56.6)	1267[a,b]	(52.6)	96	(4.0)	censored linear regression
log(cord PCB 180)	1423	(59.1)	1267[a,b]	(52.6)	156	(6.5)	censored linear regression
log(cord ΣPCBs)	1502	(62.4)	1267[a,b]	(52.6)	235	(9.8)	passive imputation
**Covariates**							
Maternal height	1	(0.0)	1	(0.0)	0	(0.0)	linear regression
Paternal height	21	(0.9)	21	(0.9)	0	(0.0)	linear regression
log(maternal BMI)	1	(0.0)	1	(0.0)	0	(0.0)	linear regression
log(paternal BMI)	45	(1.9)	45	(1.9)	0	(0.0)	linear regression
Maternal age	1	(0.0)	1	(0.0)	0	(0.0)	linear regression
Zone of residence	8	(0.3)	8	(0.3)	0	(0.0)	logistic regression
Country of birth	4	(0.2)	4	(0.2)	0	(0.0)	multinomial logistic regression
Education	5	(0.2)	5	(0.2)	0	(0.0)	ordered logistic regression
Employment during pregnancy	0	(0.0)	0	(0.0)	0	(0.0)	-
Socio-economic status	1	(0.0)	1	(0.0)	0	(0.0)	ordered logistic regression
Parity	2	(0.1)	2	(0.1)	0	(0.0)	logistic regression
Consumption of tobacco	65	(2.7)	65	(2.7)	0	(0.0)	logistic regression
Passive smoking	77	(3.2)	77	(3.2)	0	(0.0)	logistic regression
Season of last menstrual period	0	(0.0)	0	(0.0)	0	(0.0)	-

Outcome variables[a]	Imputed		Missing		Censored		Method
	n	(%)	*n*	(%)	*n*	(%)	
Sex of fetus	4	(0.2)	4	(0.2)	0	(0.0)	-
Intake of vegetables, fruit, lean fish, oily fish, and other seafood, and total energy intake during pregnancy	0	(0.0)	0	(0.0)	0	(0.0)	-
Alcohol intake during first trimester	22	(0.9)	22	(0.9)	0	(0.0)	logistic regression
log(lipids maternal serum)	241	(10.0)	241	(10.0)	0	(0.0)	linear regression
log(lipids cord serum)	1330	(55.3)	1330[b]	(55.3)	0	(0.0)	linear regression
GWG	83	(3.4)	83	(3.4)	0	(0.0)	ordered logistic regression
**Other variables (not used in main analysis)**							
log(Total mercury in cord blood)	661	(27.5)	577	(24.0)	84	(3.5)	censored linear regression
Intake of proteins, carbohydrates, fat, and caffeine during pregnancy	0	(0.0)	0	(0.0)	0	(0.0)	-
Maternal urinary cotinine (>50 ng/ml)	226	(9.4)	226	(9.4)	0	(0.0)	logistic regression
Season of maternal blood	29	(1.2)	29	(1.2)	0	(0.0)	multinomial logistic regression
Season of cord blood	3	(0.1)	3	(0.1)	0	(0.0)	multinomial logistic regression

AC: abdominal circumference; BMI: body mass index; BPD: biparietal diameter; DDE: dichlorodiphenyldichloroethylene; EFW: estimated fetal weight; FL: femur length; GWG: gestational weight gain; HCB: hexachlorobenzene; OC: organochlorine compound; PCB: polychlorinated biphenyl.

[a]In order to define a single imputed dataset (n=2407), missing values in the outcome and exposure variables (maternal and cord serum) were multiple-imputed. Subsequently, these values were deleted before analysis and recombination.

[b]Cord blood concentrations were only available for cohorts from Asturias, Gipuzkoa and Valencia.

Table S3. Pearson's correlations between OCs in maternal and umbilical cord serum. The INMA Project, 2003-2008 (Spain).

		**Maternal serum**				**Cord serum**			
		**4,4´-DDE**	**HCB**	**PCB 138**	**PCB 153**	**4,4´-DDE**	**HCB**	**PCB 138**	**PCB 153**
**ng/mL**	**HCB**	0.19	1			0.32	1		
	**PCB 138**	0.27	0.53	1		0.35	0.60	1	
	**PCB 153**	0.22	0.55	0.88	1	0.38	0.62	0.78	1
	**PCB 180**	0.13	0.54	0.81	0.85	0.31	0.54	0.76	0.82
**ng/g**	**HCB**	0.18	1			0.34	1		
	**PCB 138**	0.25	0.51	1		0.37	0.62	1	
	**PCB 153**	0.21	0.54	0.88	1	0.39	0.63	0.78	1
	**PCB 180**	0.12	0.53	0.80	0.85	0.32	0.55	0.77	0.82

DDE: dichlorodiphenyldichloroethylene; HCB: hexachlorobenzene; OC: organochlorine compound; PCB: polychlorinated biphenyl.

Number of maternal and cord pairs in ng/mL: 2369 and 1140, and in ng/g lipid: 2146 and 1077, respectively. *p*-values were <0.001 in all Pearson's correlations (adjusted by cohort).

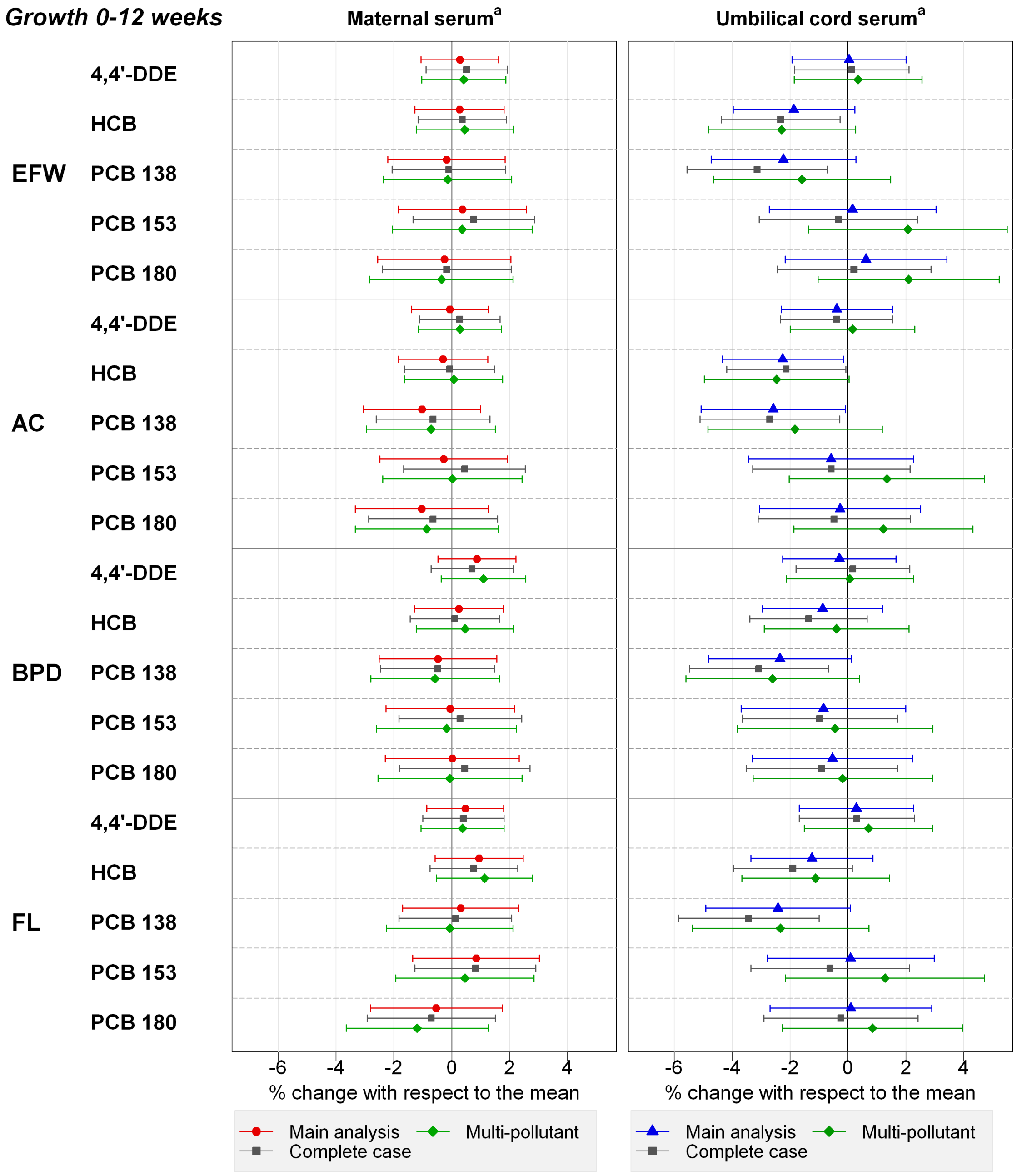

Figure S5. Sensitivity analyses of the associations between OC concentrations and fetal growth measurements between 0-12 weeks of gestation. The INMA Project, 2003-2008 (Spain).

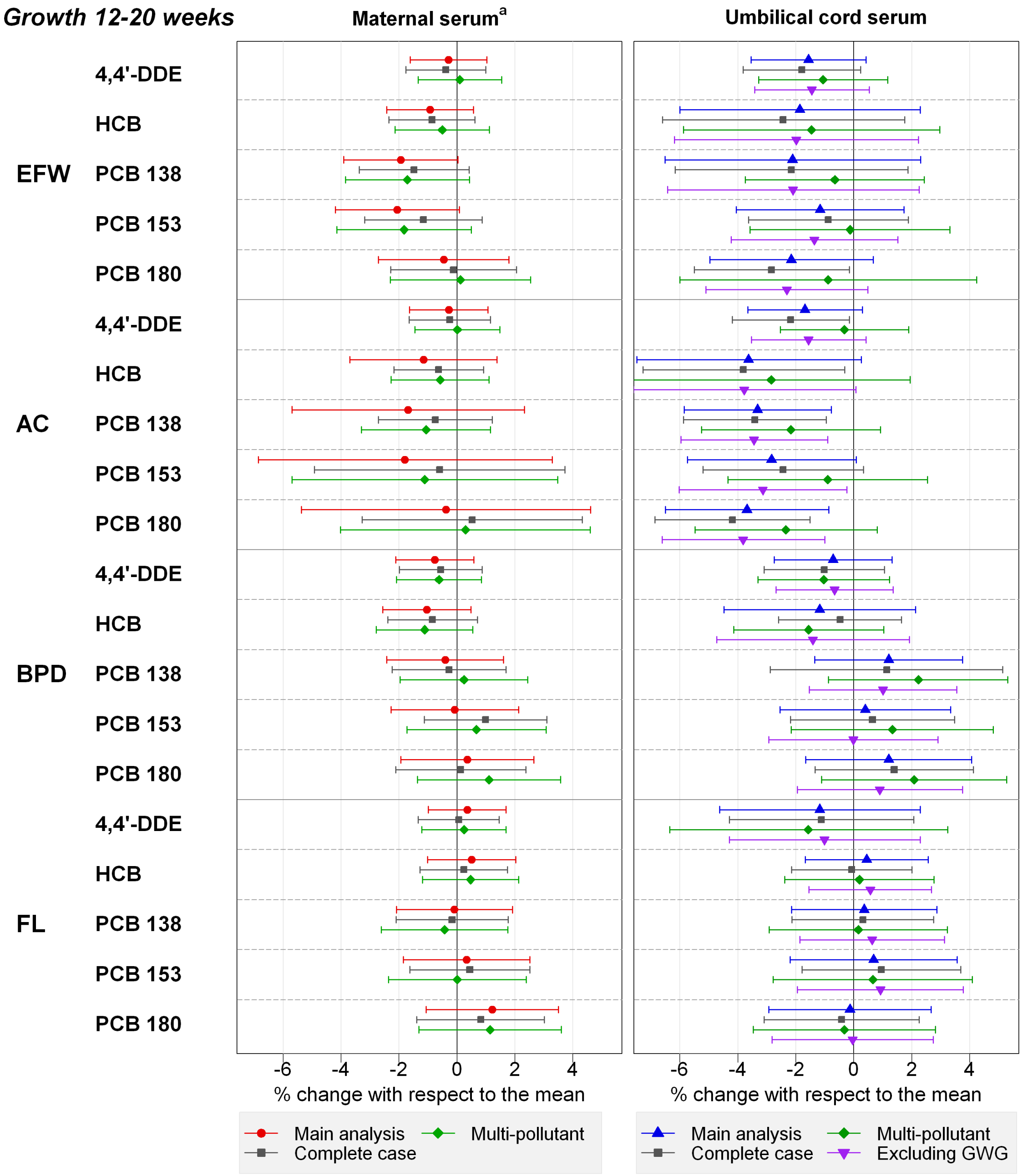

Figure S6. Sensitivity analyses of the associations between OC concentrations and fetal growth measurements between 12-20 weeks of gestation. The INMA Project, 2003-2008 (Spain).

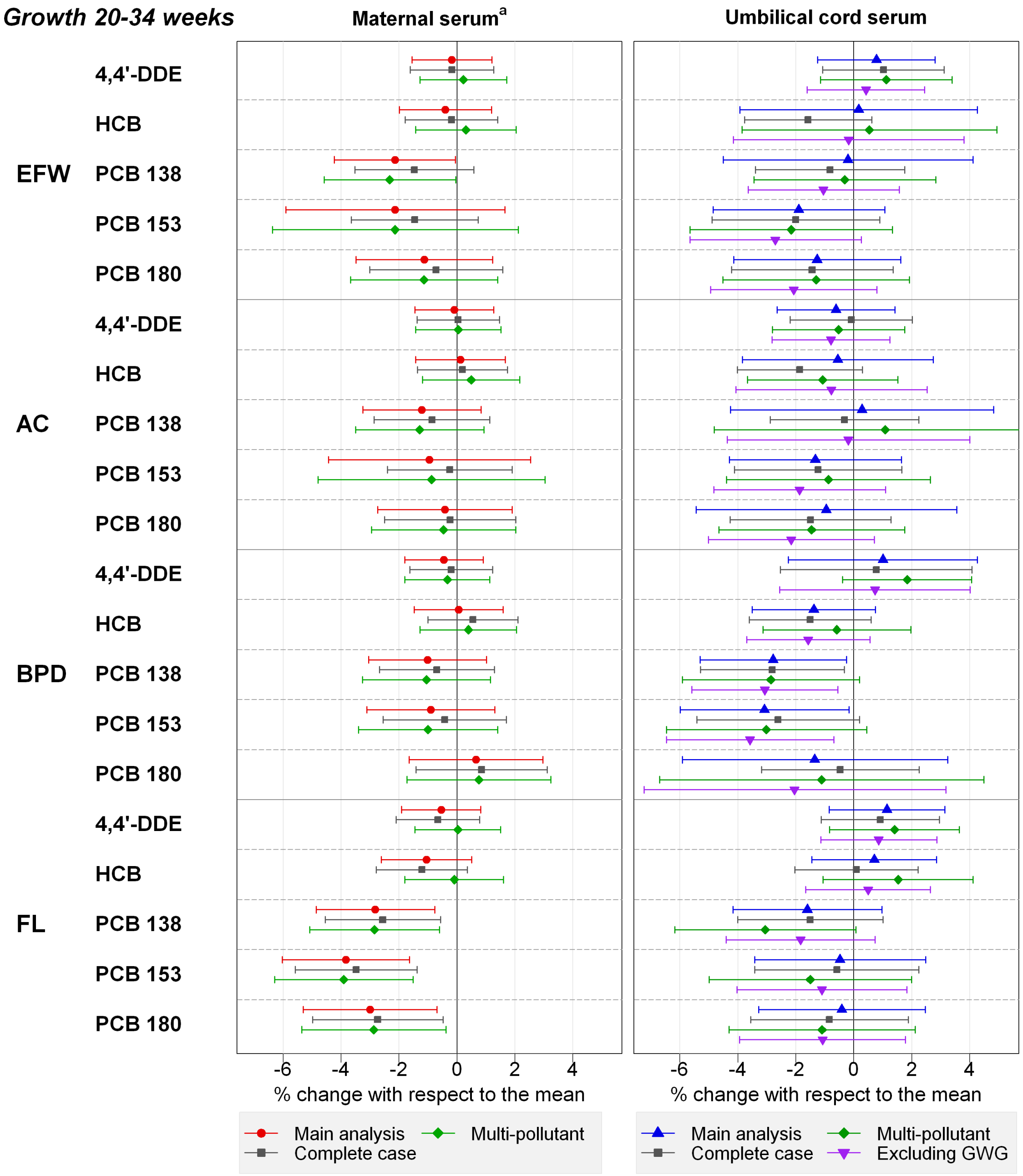

Figure S7. Sensitivity analyses of the associations between OC concentrations and fetal growth measurements between 20-34 weeks of gestation. The INMA Project, 2003-2008 (Spain).

Footnote of the Supplemental Material, Figures S5-S7

AC: abdominal circumference; BPD: biparietal diameter; DDE: dichlorodiphenyldichloroethylene; EFW: estimated fetal weight; FL: femur length; GWG: gestational weight gain; HCB: hexachlorobenzene; OC: organochlorine compound; PCB: polychlorinated biphenyl.

Adjusted linear regression models between $\log_2$(OC) concentrations and fetal growth measurements. Meta-analysis of results from multiple imputation. Results expressed as %change in fetal measurements associated with a doubling in OC concentrations.

Main analysis: results from multiple imputation; Complete case: analysis excluding cases with missing values in covariates and fixed imputation of LOD/2 for OC values <LOD; Multi-pollutant: main analysis including the OCs showing an association with fetal growth in the present analysis, i.e. models of 4,4´-DDE were additionally adjusted for ∑PCBs and HCB, models of HCB were adjusted for ∑PCBs, and models of PCBs were adjusted for HCB; Excluding GWG: analysis excluding gestational weight gain.

[a] GWG was not included in models of maternal OCs and outcomes measured at week 12 since GWG was calculated from week 12 to delivery.

References

Graham JW. 2009. Missing data analysis: making it work in the real world. Annu Rev Psychol 60:549-76.

Gurrin LC, Blake KV, Evans SF, Newnham JP. 2001. Statistical measures of foetal growth using linear mixed models applied to the foetal origins hypothesis. Stat Med 20:3391-3409.

Horton NJ, Kleinman KP. 2007. Much ado about nothing: a comparison of missing data methods and software to fit incomplete data regression models. Am Stat 61:79-90.

Lubin JH, Colt JS, Camann D, Davis S, Cerhan JR, Severson RK, et al. 2004. Epidemiologic evaluation of measurement data in the presence of detection limits. Environ Health Perspect 112:1691-1696.

Mamelle N, Cochet V, Claris O. 2001. Definition of fetal growth restriction according to constitutional growth potential. Biol Neonate 80:277-285.

Pinheiro JC, Bates DM. 2000. Mixed-effects models in S and S PLUS. Statistics and computing. New York:Springer-Verlag Inc.

R Core Team. 2014. R: A language and environment for statistical computing. Vienna, Austria: R Foundation for Statistical Computing. Available: http://www.R-project.org/. [accessed 1 December 2014].

Royston P. 1995. Calculation of unconditional and conditional reference intervals for foetal size and growth from longitudinal measurements. Stat Med 14:1417-1436.

Royston P, Altman DG. 1994. Regression Using Fractional Polynomials of Continuous Covariates: Parsimonious Parametric Modelling. Applied Statistics 43:429-467.

Van Bauren S, Groothuis-Oudshoorn K. 2011. Mice: Multivariate Imputation by Chained Equations in R. Journal of Statistical Software 45:1-67.

von Hippel PT. 2007. Regression with missing Y's: an improved method for analyzing multiply imputed data. Sociol Methodology 37:83-117.
